# PERCEPTIONS OF HOME HEALTH AIDES (HHA) CARING FOR PERSONS LIVING WITH DEMENTIA (PLWD) ABOUT ADOPTION OF TELEHEALTH

**DOI:** 10.1093/geroni/igad104.1199

**Published:** 2023-12-21

**Authors:** Zainab Toteh Osakwe, Michelle Odlum, Amarilis Céspedes, Sasha Perez

**Affiliations:** Adelphi University, Garden City, New York, United States; The George Washington University, Washington, District of Columbia, United States; Columbia University School of Nursing, New York City, New York, United States; Icahn School of Medicine at Mount Sinai, New York City, New York, United States

## Abstract

Home healthcare agencies serve a large and growing population of Persons living with Dementia (PLWD). The COVID-19 pandemic led to rapid adoption and use of telehealth services in the delivery of home healthcare, allowing patients to visit with home healthcare clinicians remotely using virtual technology. Yet, concerns remain about feasibility of telehealth for PLWD on HHC, who largely depend on home health aides to communicate their care concerns with the health care teams. We interviewed 25 HHAs to PLWD between June and August 2020 about their experiences with telehealth as part of a larger study focused on HHA informed care plans for PLWD. Interviews were recorded, transcribed, translated and analyzed using conventional content analysis. We interviewed 25 HHAs employed by 4 unique home healthcare agencies. HHAs had a mean age of 49.8 (± 9.1), 24 (97%) female, 11 (44%) Black, 12 (48%) Hispanic. Twelve HHAs were interviewed in Spanish. The main themes identified were: (1) preference for telehealth platforms that include video conferencing to enhance human interactions, (2) preference for telehealth as a supplement, not substitute, for nursing visits, (3) additional burden of using the tablet or computer while care for PLWD and (4) concerns about inadequate training needed to use telehealth. HHAs expressed concerns about the need to not substitute in person home visits with Telehealth. As home healthcare agencies consider best approaches to integrate telehealth into their care delivery models, communication with HHAs remains key to develop strategies that prioritize the care needs of PLWD.

